# Mechanism of Testosterone Deficiency in the Transgenic Sickle Cell Mouse

**DOI:** 10.1371/journal.pone.0128694

**Published:** 2015-05-29

**Authors:** Biljana Musicki, Yuxi Zhang, Haolin Chen, Terry R. Brown, Barry R. Zirkin, Arthur L. Burnett

**Affiliations:** 1 The James Buchanan Brady Urological Institute and Department of Urology, The Johns Hopkins School of Medicine, Baltimore, Maryland, United States of America; 2 Department of Biochemistry and Molecular Biology, The Johns Hopkins Bloomberg School of Public Health, Baltimore, Maryland, United States of America; Clermont Université, FRANCE

## Abstract

Testosterone deficiency is associated with sickle cell disease (SCD), but its underlying mechanism is not known. We investigated the possible occurrence and mechanism of testosterone deficiency in a mouse model of human SCD. Transgenic sickle male mice (Sickle) exhibited decreased serum and intratesticular testosterone and increased luteinizing hormone (LH) levels compared with wild type (WT) mice, indicating primary hypogonadism in Sickle mice. LH-, dbcAMP-, and pregnenolone- (but not 22-hydroxycholesterol)- stimulated testosterone production by Leydig cells isolated from the Sickle mouse testis was decreased compared to that of WT mice, implying defective Leydig cell steroidogenesis. There also was reduced protein expression of steroidogenic acute regulatory protein (STAR), but not cholesterol side-chain cleavage enzyme (P450scc), in the Sickle mouse testis. These data suggest that the capacity of P450scc to support testosterone production may be limited by the supply of cholesterol to the mitochondria in Sickle mice. The sickle mouse testis exhibited upregulated NADPH oxidase subunit gp91phox and increased oxidative stress, measured as 4-hydroxy-2-nonenal, and unchanged protein expression of an antioxidant glutathione peroxidase-1. Mice heterozygous for the human sickle globin (Hemi) exhibited intermediate hypogonadal changes between those of WT and Sickle mice. These results demonstrate that testosterone deficiency occurs in Sickle mice, mimicking the human condition. The defects in the Leydig cell steroidogenic pathway in Sickle mice, mainly due to reduced availability of cholesterol for testosterone production, may be related to NADPH oxidase-derived oxidative stress. Our findings suggest that targeting testicular oxidative stress or steroidogenesis mechanisms in SCD offers a potential treatment for improving phenotypic changes associated with testosterone deficiency in this disease.

## Introduction

Sickle cell disease (SCD) is a hemoglobinopathy resulting from the expression of abnormal sickle hemoglobin (HbS) [1, 2]. HbS arises from a mutation in both beta globin genes that substitutes a charged amino acid glutamic acid for uncharged valine. This results in aggregation of HbS molecules under deoxygenated conditions and red blood cell rigidity, leading to poor blood flow with vaso-occlusion, tissue hypoxia, and ischemia [1, 2]. In addition to hemoglobinopathy and red blood cell sickling, SCD features an independent spectrum of vascular dysfunction that involves such abnormalities as defects in nitric oxide bioavailability, abnormal interactions between sickled red blood cells, endothelial cells, platelets, and leukocytes, and elevated oxidative stress [3, 4]. SCD leads to multi-organ damage that often results in stroke, retinopathy, pulmonary hypertension, chronic kidney disease, and priapism [5].

Several clinical studies have demonstrated decreased serum testosterone levels in male patients with SCD [6–12], with rates of testosterone deficiency as high as 25% [13]. Testosterone deficiency in SCD is associated with retardation of physical development [12], infertility [14], bone mass loss [7], and possibly priapism [13]. The mechanism underlying testosterone deficiency in SCD is not clear, as both increased and decreased luteinizing hormone (LH) and follicle-stimulating hormone (FSH) levels have been measured in SCD patients. Elevated LH and FSH levels observed in patients with SCD suggests that testicular failure (primary hypogonadism) underlies dysfunctional testosterone production [6, 10, 11]. In contrast, decreased testosterone levels in SCD patients with decreased LH and FSH suggest the association of secondary hypogonadism (hypothalamo–pituitary dysfunction) with SCD [8, 15].

Given the conflicting basis for testosterone deficiency in SCD, herein we used a mouse model of human SCD to investigate the mechanism for this condition. We evaluated the possible occurrence of testosterone deficiency and the defect in the steroidogenic pathway at the levels of the whole testis and isolated Leydig cells. We further evaluated whether oxidative stress is associated with this condition. An improved understanding of the mechanism involved in SCD-associated testosterone deficiency may offer approaches to address this condition in men with SCD.

## Materials and Methods

### Chemicals and Reagents

Dibutyryl cAMP (dbcAMP) and 22-hydroxycholesterol were obtained from Sigma-Aldrich. Pregnenolone was obtained from Steraloids (purity 99.8%). Testosterone antibody was obtained from Fitzgerald Industries International. Bovine LH (USDA-bLH-B-6) was provided by the United States Department of Agriculture Animal Hormone Program [16].

Polyclonal rabbit anti-cytochrome P450scc cholesterol side-chain cleavage enzyme (P450scc) antibody was obtained from Millipore (#ABS235). Polyclonal rabbit anti-steroidogenic acute regulatory protein (StAR) antibody was obtained from Santa Cruz Biotechnology (#25806). Monoclonal mouse anti-gp91^phox^ antibody was obtained from BD Transduction Laboratories (#611414). Polyclonal rabbit anti-4-hydroxy-2-nonenal (HNE) antibody was obtained from Alpha Diagnostic International (#HNE-11S). Polyclonal rabbit anti-glutathione peroxidase-1 (GPx-1) antibody was obtained from Abcam (#22604). Monoclonal mouse anti β-actin antibody was obtained from Sigma Chemical (#A5316). The horseradish peroxidase-conjugated anti-mouse (#NA931V) or anti-rabbit (#NA934V) donkey second antibodies were obtained from GE Healthcare Biosciences.

### Mouse Model of Human SCD

Transgenic sickle mice (Sickle) with knockout of all mouse hemoglobin genes and insertion of a transgene that expresses human α- and β HbS were developed at Lawrence Berkeley National Laboratory [17]. Sickle mice were obtained by interbreeding sickle cell males with hemizygous (Hemi) females in-house. Breeding pairs for Sickle mice (strain number 3342) and wild type (WT) mice were obtained from Jackson Laboratory. Genotyping was performed by Transnetyx, Inc. Because C57BL/6 is one of the background strains for the transgenic sickle mice, C57BL/6 was chosen as WT control. Additional control animals were Hemi littermates, which have anemia but no sickle deformation [18]. Male mice used in experiments were 7–9 months old. Mice were pathogen free and received routine NIH rodent chow and water. Testes and blood were collected between 8 am and 11 am from anesthetized mice. Blood was obtained by cardiac puncture using 1 ml TB syringe with sterile 26Gx3/8 needle and collected into eppendorf tubes. Although we did not measure fetal Hb or erythrocyte sickling in Sickle mice, we did confirm their phenotype after euthanasia by their extremely big spleens [17].

### Ethics Statement

All animal procedures were approved by the Johns Hopkins University School of Medicine Animal Care and Use Committee. Mice were anesthetized with 50 mg/kg Ketamine + 5 mg/kg Xylazine by intraperitoneal injection. All efforts were made to minimize animal suffering.

### Blood Sampling and Hormone Measurements

After collection as above, blood was kept for 1 h at room temperature for LH measurements, or at 4 C for testosterone measurements. All test tubes were centrifuged at 3,000 x g for 10 minutes, and serum was separated. Serum LH was measured at the University of Virginia, Center for Research in Reproduction, by immunoradiometric assay [19]. Serum and intratesticular testosterone were measured by radioimmunoassay (RIA) [20].

### Leydig Cell Quantification, Isolation and Culture

Leydig cells were isolated by a combination of Percoll and BSA density gradient centrifugations, as previously described [21]. Briefly, the testes were decapsulated and digested in a dissociation buffer (M-199 medium with 2.2 g/l HEPES, 1.0 g/l BSA, 2.2 g/l sodium bicarbonate) containing collagenase I (0.5 g/l) at 34°C with slow shaking (90 cycles/min, 30 minutes). To separate the interstitial cells from the seminiferous tubules, digested testes were mixed with M199 containing 10% BSA so the final concentration of BSA reached 1%. The mixed solution containing digested cells and tissue was incubated for 1 minute to separate single cells and small cell clusters from large seminiferous tubules. The supernatant was transferred into 15 ml centrifuge tubes (Sarstedt Inc, Newton, NC; #62.554.002), and the cells were pelleted by centrifugation (250 x g, 5 min). The pellet was resuspended in 5 ml of Percoll separation solution (55% Percoll in Hank’s Balance salts solution with 1.5 mM HEPES and 0.025% BSA) and transferred to an Oakridge centrifuge tubes (#05-563-2C). After adding 30 ml Percoll separation solution to the tube and mixing, the tube was centrifuged (27,000 x g, 60 min) at 4 C to generate a continuous density gradient. A fraction containing Leydig cells (about 10 ml) was collected from the gradient layer that was heavier than 1.068 g/ml density. The Leydig cell fraction was transferred into a 50 ml centrifuge tube, diluted with DMEM/F12 medium and pelleted by centrifugation (250 x g, 5 min). The pellet was then dissolved in 1 ml DMEM/F12 medium and layered on top of a BSA separation gradient formed in a 15-ml tube (containing 3 ml each of, top to bottom, 2.5%, 5%, 10% BSA in DMEM/F12 medium). The tube was centrifuged at 4 C (50 x g, 10 min). The pellet was suspended and washed in 5 ml DMEM/F12 medium and centrifuged (250 x g, 5 min). The final cell pellet was resuspended in 0.5 ml M-199 culture medium and counted. The final purity of the Leydig cells, determined by staining the cells for 3β-hydroxysteroid dehydrogenase (3β-HSD) activity, was consistently about 90% [20].

Freshly isolated Leydig cells were suspended in M-199 culture media (0.1% BSA), plated in 96-well culture plates (about 10^4^ cells/well), and incubated for 2h at 34°C with steroidogenesis effectors bovine LH (0.5 ng/ml and 10 ng/ml), dbcAMP (1 mM), 22-hydroxycholesterol (25 μM), or pregnenolone (25 μM). After incubation, cells plus medium were frozen on dry ice and then stored at −80°C for testosterone assay by RIA.

### Western Blot Analysis

Testes were homogenized as previously described [22]. Protein concentration was determined using bicinchonic acid method with BSA as a standard. Homogenates (30–50 μg) were resolved on 4–20% Tris gels and transferred to polyvinylidene difluoride membranes. Membranes were blocked for 1 hour at room temperature in PBS (pH 7.4) containing 0.1% Tween-20 and 5% nonfat dry milk, and then probed overnight with anti-P450scc antibody at 1:6,000 dilution, anti-StAR antibody at 1:3,000 dilution, anti-gp91^phox^ (a catalytic subunit of reactive oxygen species-generating enzyme NADPH oxidase, [23]) antibody at 1:1,000 dilution, anti-GPx-1 (an antioxidant enzyme, [24]) antibody at 1:1,000 dilution, and anti-4-HNE (a marker of oxidative stress, [25]), antibody at 1:5,000 dilution. Signals were standardized to β-actin (1:7,000 dilution) [26]. Bands were detected by horseradish peroxidase conjugated anti-mouse or anti-rabbit secondary antibodies and quantified using NIH Image 1.29 (National Institutes of Health). To verify that β-actin expression was not affected by SCD, the density of a set of samples was standardized per total proteins (Ponceau S staining). Ratios did not differ from those obtained using β-actin for standardization (data not shown).

### Statistical Analysis

Statistical analyses were performed using one-way analysis of variance (ANOVA; SigmaStat Windows Version 3.00; Systat Software), followed by Newman-Keuls multiple comparison test (for serum testosterone, LH and for Leydig cell testosterone measurements). Intratesticular testosterone levels were analyzed by Mann Whitney (nonparametric) test due to small sample size. For Western blots, we used a modified t test to compare the experimental groups with the normalized control ratio. The data were expressed as the mean ± standard error of the mean (SEM). A value of P < 0.05 was considered to be statistically significant. The original data of the article are in S1 Dataset.

## Results

### Body and Testicular Weights

Body weights did not differ significantly (P>0.05) among WT (31.5 ± 0.5 g), Hemi (34.3 ± 1.8 g), and Sickle (29.9 ± 1.0 g) mice. Testis weights (WT, 206.3 ± 4.2 mg; Hemi, 235.2 ± 4.3 mg; Sickle, 209.4 ± 7.3 mg) also did not differ (P>0.05).

### Decreased Serum and Intratesticular Testosterone Concentrations in Sickle Mice

**Fig 1:**
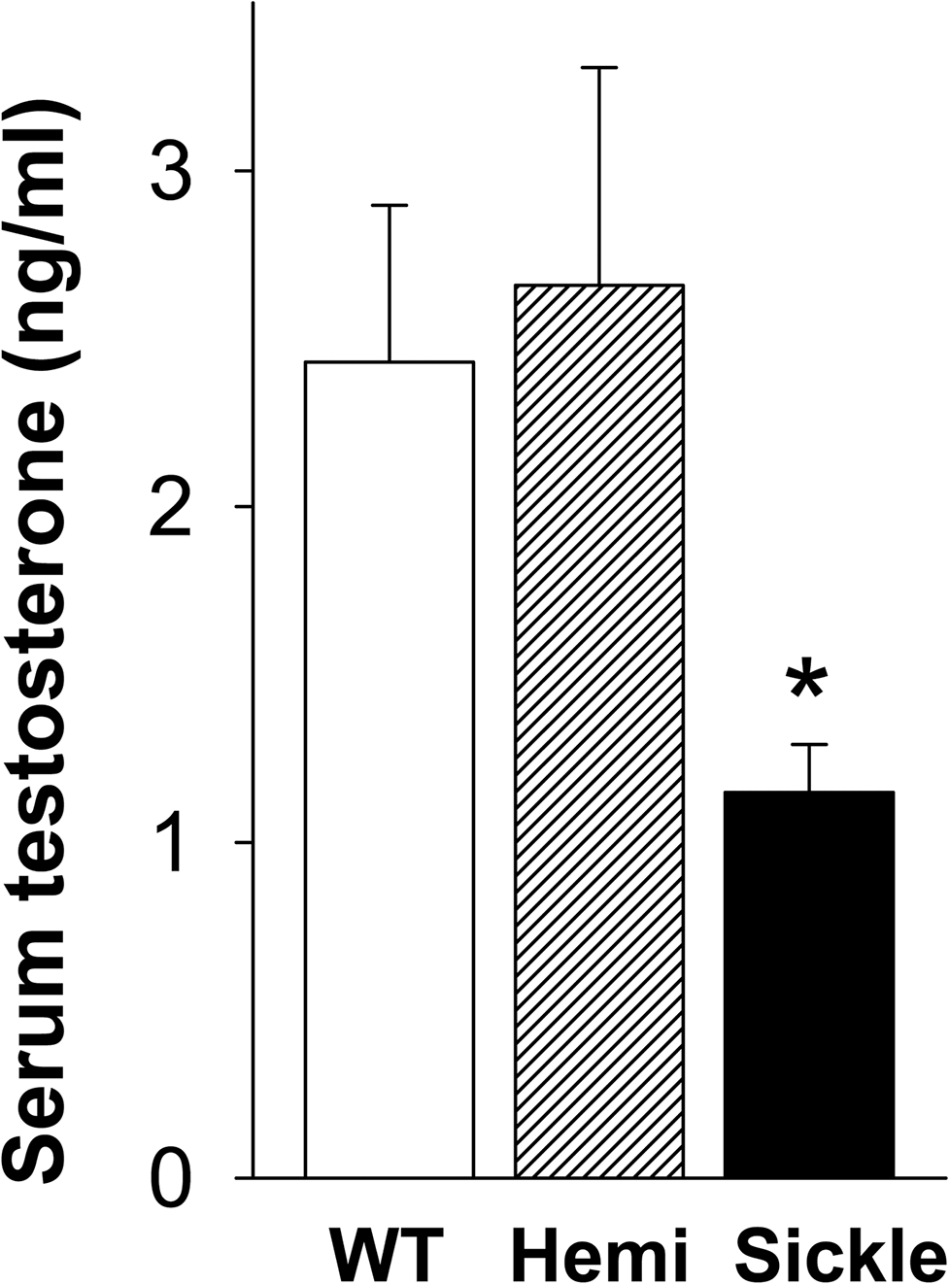
Serum testosterone levels are decreased in Sickle mice. Testosterone levels in WT, Hemi, and Sickle mice were measured by RIA. Each bar represents the mean ± SEM. *P < 0.05 vs WT and Hemi. n = 10–30.

**Fig 2:**
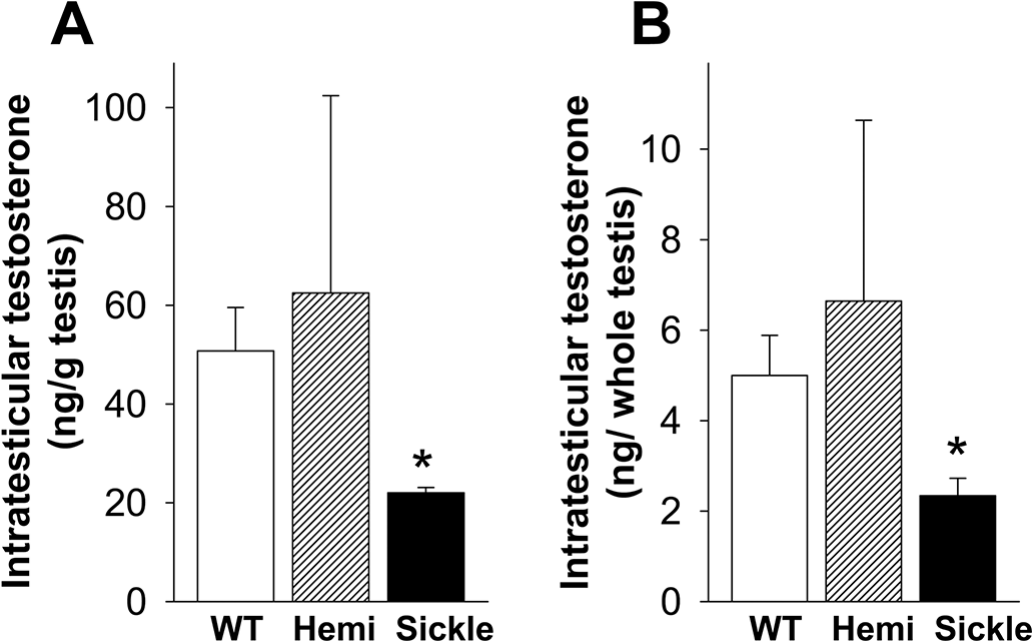
Intratesticular testosterone levels are decreased in Sickle mice. Intratesticular testosterone levels, expressed per gram testis (A) and per whole testis (B), in WT, Hemi, and Sickle mice, were measured by RIA. Each bar represents the mean ± SEM. *P < 0.05 vs WT. n = 4–6.

Serum testosterone levels were significantly lower in the Sickle mice as compared to WT and Hemi mice (P<0.05, Fig 1). Consistent with serum testosterone levels, intratesticular testosterone levels, whether expressed per gram testis or per testis, were significantly reduced in Sickle mice compared to WT mice (P<0.05) (Fig 2). Intratesticular testosterone levels were highly variable in the Hemi mice and so did not differ significantly (P>0.05) from the Sickle mice. However, there was a 2-3-fold difference in the means. These findings indicate that testosterone deficiency occurs in Sickle mice.

### Increased Serum LH Levels in Sickle Mice

**Fig 3:**
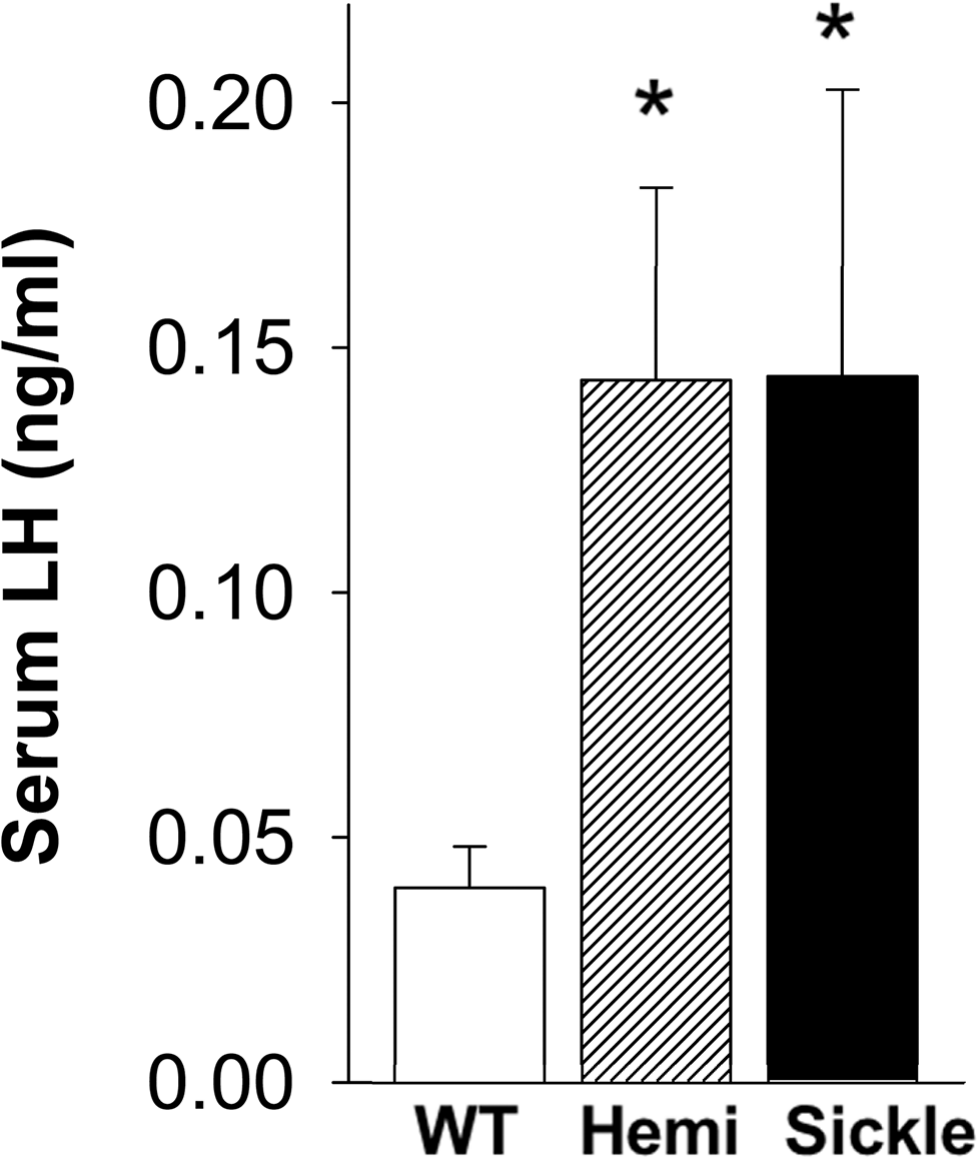
Serum LH Levels are increased in Sickle and Hemi mice. Serum LH levels in WT, Hemi, and Sickle mice were measured by immunoradiometric assay. Each bar represents the mean ± SEM. *P < 0.05 vs WT. n = 18–29.

Serum LH levels were significantly higher in Sickle and Hemi mice as compared to WT mice (P<0.05, Fig 3). These results indicate derangement in the hypothalamic-pituitary axis in Sickle mice.

### Impaired Testosterone Production by Leydig Cells in Sickle Mice

**Fig 4:**
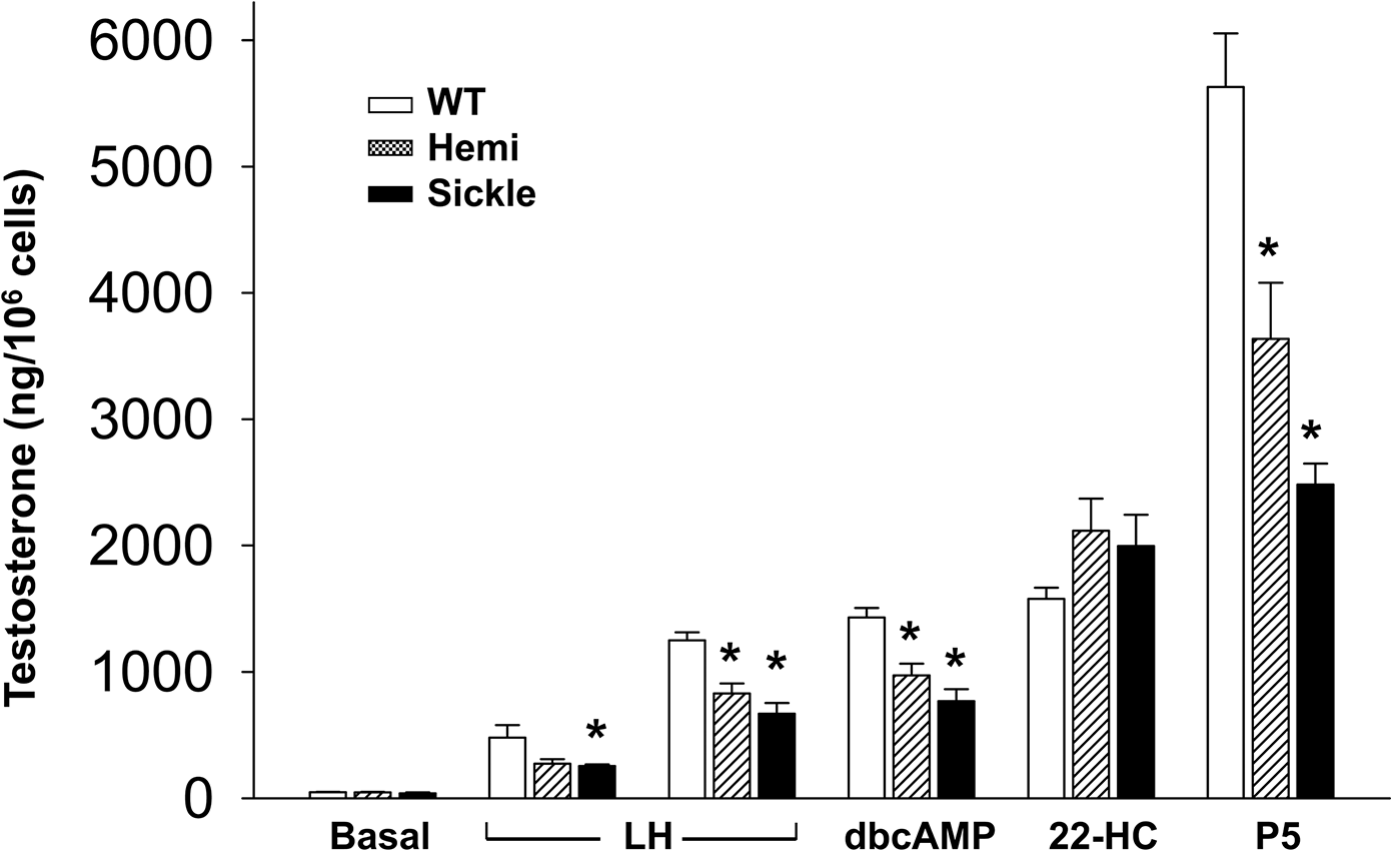
Testosterone production by Sickle and Hemi mouse Leydig cells is impaired. In vitro testosterone production from Leydig cells isolated from WT, Hemi, and Sickle mouse testis. Cells were stimulated, or not, with LH (0.5 ng/ml and 10 ng/ml), dbcAMP (1 mM), 22-hydroxycholesterol (25 μM; 22-HC), and pregnenolone (25 μM, P5). Each bar represents the mean ± SEM. *P < 0.05 vs WT. n = 7–13.

We hypothesized that differences in the activities of enzymes of the steroidogenic pathway likely account for decreased serum and intratesticular testosterone production by Leydig cells of Sickle mice. To determine whether this is the case, Leydig cells isolated from WT, Hemi and Sickle mice were incubated for 2 hours with steroiodogenic effectors LH, dbcAMP, 22-hydroxycholesterol, or pregnenolone, and testosterone production was measured (Fig 4). Incubation of the cells with LH, dbcAMP, 22-hydroxycholesterol, or pregnenolone resulted in each case in significant (P<0.05) stimulation of testosterone production over baseline. However, with each of LH, dbcAMP and pregnenolone, testosterone production by Leydig cells isolated from the Sickle and Hemi mice was significantly (P<0.05) lower than that by WT cells, whereas incubation of the cells with 22-hydroxycholesterol resulted in equivalent stimulation of the cells.

### Decreased Protein Expression of StAR but not P450scc in the Testis of Sickle Mice

**Fig 5:**
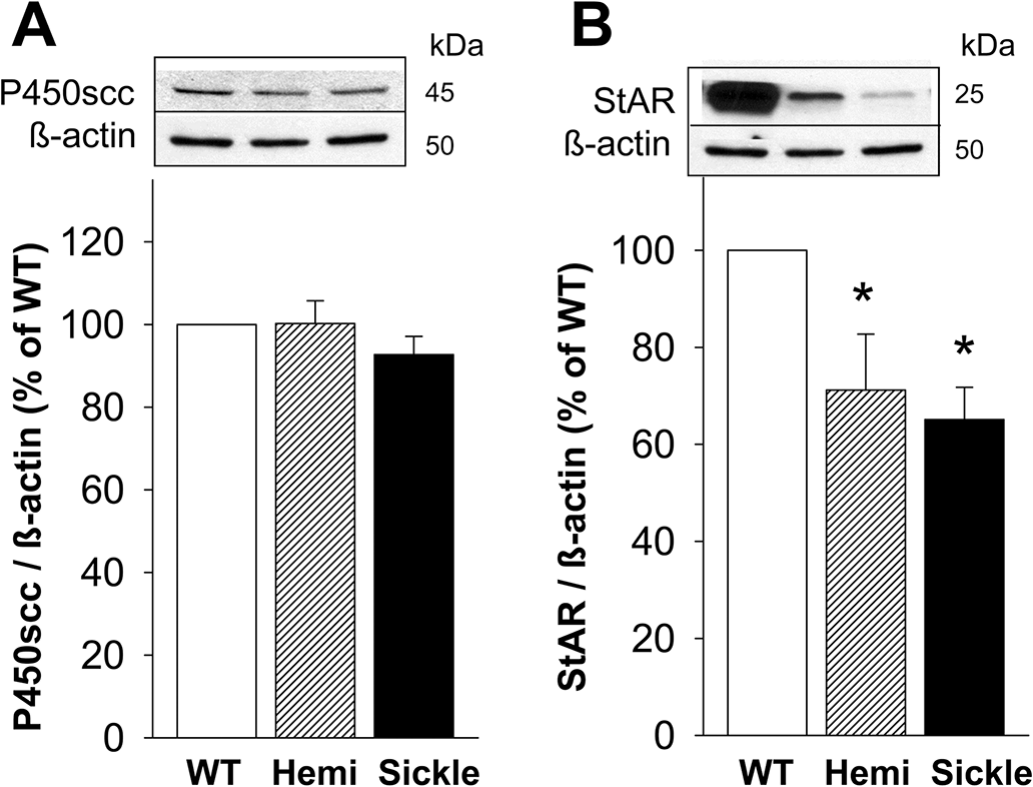
Protein expression of StAR, but not P450scc, is decreased in the testis of Sickle and Hemi mice. Protein expressions of P450scc (A) and StAR (B) in the testis of WT, Hemi, and Sickle mice were measured by Western immunoblot. Upper panels are representative Western immunoblots. Lower panels represent quantitative analyses of the proteins in the same groups. Each bar represents the mean ± SEM. *P < 0.05 vs WT. n = 7–15. StAR = steroidogenic acute regulatory protein; P450scc = cholesterol side-chain cleavage enzyme.

To evaluate whether testosterone deficiency in Sickle mice involves decreased expressions of major proteins involved in steroid production, we measured protein levels of P450scc and StAR in testes of Sickle, Hemi, and WT mice by Western blot analysis. Protein expression of P450scc did not differ (P>0.05) among the Sickle, Hemi, and WT testes (Fig 5A). Protein expression of StAR was significantly (P<0.05) reduced in the testis of Sickle and Hemi mice compared to that of WT mice (Fig 5B).

### Increased Oxidative Stress Markers in the Testis of Sickle Mice

**Fig 6:**
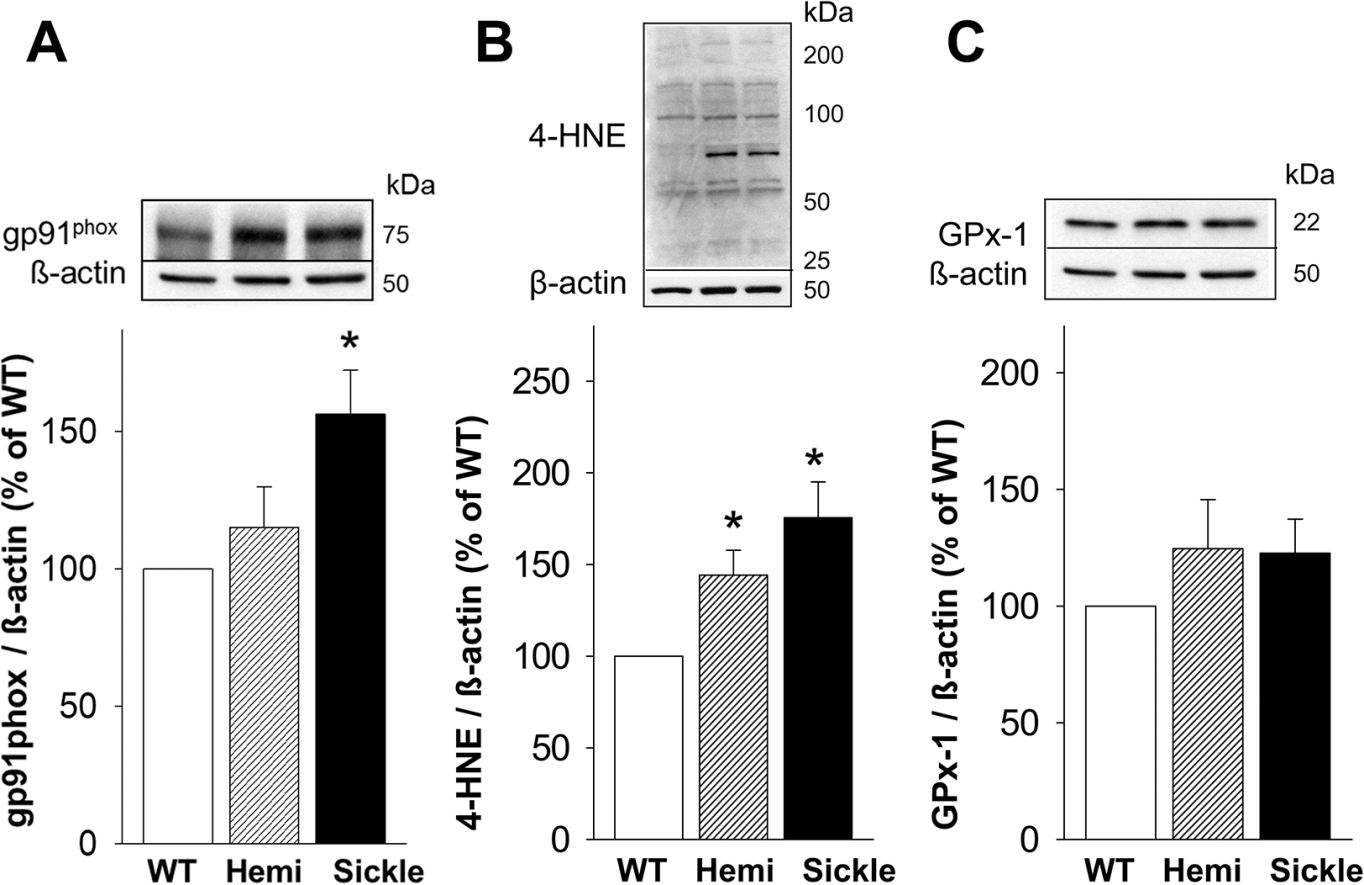
Oxidative stress markers are increased in the testis of Sickle and Hemi mice. Protein expressions of NADPH oxidase subunit gp91^phox^ (A), oxidative stress marker 4-HNE (B), and antioxidant enzyme GPx-1 (C) in the testis of WT, Hemi, and Sickle mice were measured by Western immunoblot. The analysis of 4-HNE is a densitometric composite of all proteins in each lane [26, 27]. Upper panels are representative Western immunoblots. Lower panels represent quantitative analyses of the proteins in the same groups. Each bar represents the mean ± SEM. *P < 0.05 vs WT. n = 8–16. 4-HNE = 4-hydroxy-2-nonenal; Gpx-1 = glutathione peroxidase-1.

To evaluate whether testosterone deficiency in Sickle mice involves increased oxidative stress at the testicular level, we measured the protein expression of several markers of oxidative stress in the testis of Sickle, Hemi, and WT mice by Western blot analysis:

gp91^phox^ (a catalytic subunit of reactive oxygen species-producing enzyme NADPH oxidase) [23]; 4-HNE (a major product of lipid peroxidation of polyunsaturated fatty acids, which covalently binds to cysteine, lysine, and histidine residues of proteins and is an indicator of protein peroxidation and a biomarker for oxidative damage) [25]; and GPx-1 (an antioxidant enzyme that reduces hydrogen peroxide to water and lipid peroxides to their corresponding alcohols) [24]. Protein expression of gp91^phox^ was significantly (P<0.05) increased in the testis of Sickle compared to that of WT mice, but it did not differ from that of Hemi mice (Fig 6A). The amount of 4-HNE modified proteins was significantly (P<0.05) upregulated in testes of Sickle and Hemi compared to WT mice (Fig 6B). Protein expression of GPx-1 did not differ among WT, Hemi and Sickle mouse testes (P>0.05, Fig 6C). These results suggest increased oxidative stress in the testis of Sickle mice involving upregulated NADPH oxidase. The intermediately increased expression of 4-HNE coupled with basal expression of gp91^phox^ in the testis of Hemi mice suggest lesser oxidative stress effects that do not involve NADPH oxidase activation.

## Discussion

The present study was designed to investigate whether testosterone deficiency occurs in SCD, and what pathophysiology is associated with it, using a transgenic mouse model of human SCD. We demonstrated that Sickle mice exhibited decreased serum and intratesticular testosterone levels, indicating that testosterone deficiency occurs in these mice as is also observed in patients with SCD [6–12]. We further established that the type of “hypogonadism” is primary in Sickle mice (based on elevated LH levels) and identified a possible basis for the testicular defect resulting in impaired steroidogenesis (involving reduced StAR protein expression and impaired Leydig cell function). Our further discovery that oxidative stress occurs in the Sickle testis and is related to NADPH oxidase activation offers a possible mechanism for decreased testosterone production in Sickle mice.

With the goal of determining whether reduced serum and intratesticular testosterone levels in the Sickle mice result from central changes in the hypothalamic-pituitary axis, LH levels were measured in the serum of these mice. Increased serum LH levels were seen in Sickle mice, the likely explanation for which is reduced serum testosterone and therefore reduced negative feedback on the hypothalamic-pituitary axis [28]. Reduced testosterone and elevated LH are features of classical primary hypogonadism [28]. Further studies are needed to evaluate whether age-related changes in LH levels occur in Sickle mice.

It seemed likely that the observed primary hypogonadism in Sickle mice results from defects in the Leydig cell steroidogenic pathway. To address this, we compared major components of the steroidogenic pathway in Sickle mice. Leydig cell testosterone production is principally regulated by LH. Acutely, LH regulates the rate-limiting step in testosterone production, the transfer of cholesterol, the precursor of steroids, into the inner mitochondrial membrane where the P450scc enzyme (also known as CYP11A1) converts it to pregnenolone [29]. Trophically, LH maintains the activities of multiple steroidogenic enzymes, including P450scc enzyme and those involved in converting pregnenolone to testosterone in the endoplasmic reticulum. The movement of cholesterol across the outer to the inner mitochondrial membrane requires the involvement of the transduceosome, an ensemble of mitochondrial and cytosolic proteins, including translocator protein (TSPO) and StAR, respectively [29, 30]. TSPO functions in the cholesterol trail from intracellular stores to the inner mitochondrial membrane [31]. Upon hormonal stimulation, STAR is activated and plays a role in increasing the flow of cholesterol to mitochondria, functioning at the outer mitochondrial membrane [32]. Our finding of decreased StAR protein expression in the Sickle mouse testis is thus consistent with an impaired steroidogenic function in Sickle mice.

We next localized the defect in steroidogenesis in the Sickle mouse testis. Incubation of Leydig cells isolated from Sickle mice with each of LH, dbcAMP, 22-hydroxycholesterol and pregnenolone resulted in increased testosterone formation. However, the increases in response to LH, dbcAMP and pregnenolone were less substantial in cells of the Sickle mice compared to that of WT mice, and there was no difference in testosterone production when the cells were incubated with 22-hydroxycholesterol, which by-passes cholesterol transport into the mitochondria. These observations are consistent with reduced protein expression of StAR, but not P450scc, in the Sickle mouse testis, indicating that the capacity of P450scc enzyme to support testosterone production may be limited by reduced supply of cholesterol to the mitochondria. In sum, impaired cholesterol transport in Leydig cells of Sickle mice serves as a mechanism for reduced testosterone production by these cells. Interestingly, when Leydig cells from Sickle mouse testes were stimulated with pregnenolone, which by-passes the regulatory processes in mitochondria, testosterone production also was decreased. This indicates that there also are post-mitochondrial steps in steroidogenesis that are affected in Leydig cells of Sickle mice. We propose, however, that it is the rate-determining step of steroidogenesis, cholesterol transport into the mitochondria, that is the key deficit in Leydig cells of Sickle mice that accounts for primary hypogonadism in this mouse model.

We hypothesized that the defects in the steroidogenic pathway may involve oxidative stress in the testis. Oxidative stress is known to play a significant role in SCD pathophysiology. Increased reactive oxygen species production in SCD has been attributed to activation of NADPH oxidase and xanthine oxidase, eNOS uncoupling, autooxidation of HbS, heme iron release, and increased asymmetric dimethylarginine [3]. The NADPH oxidases are a family of enzymes that catalyze electron transfer from cytosolic NADPH or NADH to molecular oxygen to generate superoxide. The prototype Nox2 (gp91^phox^)-containing NADPH oxidase possesses cytosolic subunits (p47^phox^, p67^phox^, or homologues) and membrane-bound subunits (gp91^phox^ and p22^phox^), which form a functional enzyme complex upon activation [23]. NADPH oxidases are activated by diverse stimuli such as hypoxia, angiotensin II, proinflammatory cytokines, vasoconstrictors, growth factors, metabolic factors, and superoxide itself [23].

We found upregulated NADPH oxidase subunit gp91^phox^ in the testis of Sickle mice, implying activated NADPH oxidase as a source of oxidative stress. While the mechanism for upregulation of NADPH oxidase in the Sickle mouse testis is not known, it is plausible that the testicular hypoxic state is involved. In SCD, vasoocclusive events (due to abnormal polymerization of HbS), and hemolytic anemia (due to the fragility of red blood cells), contribute to hypoxia in peripheral tissues [33, 34]. Intermittent hypoxia has been shown to induce upregulation of NADPH oxidase catalytic subunit gp91^phox^ and regulatory subunits p22^phox^ and p47^phox^ in the testis; the effect of oxidative stress decreases mRNA and protein expressions of StAR and 3β-HSD and results in hypogonadism [35]. It is plausible that NADPH oxidase-derived oxidative stress in the Sickle mouse testis inhibits steroidogenesis by interfering with cholesterol transport into mitochondria. As further evidence of increased oxidative stress in the testis of Sickle mice, protein expression of 4-HNE, a major end product of lipid peroxidation [36], was increased. 4-HNE is a relatively stable end product of lipid peroxidation used as a biomarker for oxidative damage. It is membrane diffusible and highly reactive, and by covalently binding to histidine, lysine, and cysteine residues of proteins it forms adducts that alter protein function and structure [25, 36]. GPx-1 is an antioxidant enzyme abundant in Leydig cells [37] that reduces hydrogen peroxide to water and lipid peroxides to their corresponding alcohols. Eight different isoforms of GPx have been discovered. GPx1 is the ubiquitous isoform expressed in the cytosol and mitochondria in most cells [24]. In these ways, GPx-1 counteracts oxidative stress. Surprisingly, GPx-1 was comparable in expression in Leydig cells of Sickle and WT mice. These results indicate an altered redox environment in Leydig cells of Sickle mice, with increased production of pro-oxidants and a decreased ability to counter their effects.

Hypoxia may also affect testicular microvasculature blood flow, which delivers oxygen, nutrients and hormones into testicular interstitial fluid [38, 39]. Endothelial dysfunction is central to vascular pathophysiology in SCD, resulting in reduced blood flow, vasoconstriction, activation of platelets and coagulation, and increased adhesion receptor expression on vascular endothelium [2]. The lack of adequate blood flow in the testis is reported to result in cell necrosis [40]. Erythrocyte sickling, obstructed blood flow, and hypoxia promote testicular infarction. Testicular infarction has been reported in patients with SCD, and has been proposed to contribute to testicular failure in this patient group [41–43].

Hemi mice exhibited some of the defects in the testicular steroidogenic pathway, oxidative stress, and elevated LH levels as did Sickle mice although, in contrast to the Sickle mice, they did not exhibit testosterone deficiency. The condition of normal testosterone and high LH levels, as we have shown here in Hemi mice, parallels observations in man. Studies of aging men have revealed a condition referred to as putative “compensated hypogonadism” [44, 45]. In these men, as in the Hemi mice, serum testosterone is in the normal range but LH levels are quite high. In man, it has been speculated that this condition represents subclinical hypogonadism that may eventually develop into overt primary hypogonadism. The condition is described to be analogous to subclinical hypothyroidism in which there is high thyroid stimulating hormone (TSH) produced by the pituitary, amid normal thyroid hormone levels [46]. Other studies also reported that pathologic abnormalities in Hemi mice are generally intermediate between those of control and Sickle mice, or may even be severe [47]. For example, oxidative stress was shown to be increased in the cremaster muscle of both Sickle and heterozygous mice compared with WT controls [48]. Similar to Hemi mice, humans with sickle cell trait (carrying one copy of HbS), while usually healthy, may manifest symptoms of SCD such as pain crises, hematuria, increased frequency of urinary tract infections, leg ulcers, kidney disease, and renal dysfunction to a varying degree [49, 50]. More studies are needed to elucidate mechanisms underlying phenomena observed in Hemi mice.

In conclusion, this study provides the first evidence that testosterone deficiency occurs in Sickle mice, mimicking the human condition. Testosterone deficiency in Sickle mice primarily involves defects at the testicular level. Defects in the Leydig cell steroidogenic pathway, related to reduced availability of cholesterol for testosterone production, exist coupled with NADPH oxidase-derived oxidative stress. Future studies are needed to determine whether providing testosterone precursors may alleviate some of the pathologies associated with SCD. Additionally, our findings suggest that targeting testicular oxidative stress mechanisms in SCD may have therapeutic relevance for conditions ranging from physical underdevelopment to infertility, bone mass loss, and possibly priapism.

## Supporting Information

S1 Dataset The original data of the article. (XLSX)

## References

[1] Sun K, Xia Y. New insights into sickle cell disease: a disease of hypoxia. Curr Opin Hematol. 2013;20: 215–221. 10.1097/MOH.0b013e32835f55f9 23549375

[2] Kato GJ, Hebbel RP, Steinberg MH, Gladwin MT. Vasculopathy in sickle cell disease: Biology, pathophysiology, genetics, translational medicine, and new research directions. Am J Hematol. 2009;84: 618–625. 10.1002/ajh.21475 19610078 PMC3209715

[3] Wood KC, Granger DN. Sickle cell disease: Role of reactive oxygen and nitrogen metabolites. Clin Exp Pharmacol Physiol. 2007;34: 926–932. 17645642 10.1111/j.1440-1681.2007.04639.x

[4] Akinsheye I, Klings ES. Sickle cell anemia and vascular dysfunction: The nitric oxide connection. J Cell Physiol. 2010;224: 620–625. 10.1002/jcp.22195 20578237

[5] Kassim AA, DeBaun MR. Sickle cell disease, vasculopathy, and therapeutics. Annu Rev Med. 2013;64: 451–466. 10.1146/annurev-med-120611-143127 23190149

[6] Abbasi AA, Prasad AO, Ortega J, Conego E, Oberleas D: Gonadal function abnormalities in Sickle cell anaemia. Studies in adult male patients. Ann Int Med. 1976;85: 601–605. 984611 10.7326/0003-4819-85-5-601

[7] Abudu EK, Akanmu SA, Soriyan OO, Akinbami AA, Adediran A, Adeyemo TA, et al Serum testosterone levels of HbSS (sickle cell disease) male subjects in Lagos, Nigeria. BMC Res Notes. 2011;4: 298 10.1186/1756-0500-4-298 21849076 PMC3170610

[8] Dada OO, Nduka EU. Endocrine function and Haemoglobinopathies: Relation between the sickle cell gene and circulatory levels of testosterone, LH and FSH in adult males. Clin Chim Acta. 1980;105: 269–273. 6772354 10.1016/0009-8981(80)90469-6

[9] Parshad O, Stevens MC, Preece MA, Thomas PW, Serjeant GR: The mechanism of low testosterone levels in homozygous sickle-cell disease. West Indian Med J. 1994;43(Suppl 1): 12–14. 8036809

[10] Osegbe DN, Akinyanju OO. Testicular dysfunction in men with sickle cell disease. Postgrad Med J. 1987;63: 95–98. 3118348 10.1136/pgmj.63.736.95 PMC2428240

[11] Brachet C, Heinrichs C, Tenoutasse S, Devalck C, Azzi N, Ferster A. Children with sickle cell disease: growth and gonadal function after hematopoietic stem cell transplantation. J Pediatr Hematol Oncol. 2007;29: 445–450. 17609621 10.1097/MPH.0b013e31806451ac

[12] Singhal A, Gabay L, Serjeant GR. Testosterone deficiency and extreme retardation of puberty in homozygous sickle-cell disease. West Indian Med J. 1995;44: 20–23. 7793108

[13] Morrison BF, Reid M, Madden W, Burnett AL. Testosterone replacement therapy does not promote priapism in hypogonadal men with sickle cell disease: 12‐month safety report. Andrology. 2013;1: 576‐582. 10.1111/j.2047-2927.2013.00084.x 23606509

[14] Modebe O, Ezeh UO. Effect of age on testicular function in adult males with sickle cell anemia. Fertil Steril. 1995;63: 907–912. 7890081 10.1016/s0015-0282(16)57500-1

[15] el-Hazmi MA, Bahakim HM, al-Fawaz I. Endocrine functions in sickle cell anaemia patients. J Trop Pediatr. 1992;38: 307–313. 1844090 10.1093/tropej/38.6.307

[16] Chandrashekar V, Bartke A, Wagner TE. Interactions of human growth hormone and prolactin on pituitary and Leydig cell function in adult transgenic mice expressing the human growth hormone gene. Biol Reprod. 1991; 44:135–140. 2015345 10.1095/biolreprod44.1.135

[17] Pászty C, Brion CM, Manci E, Witkowska HE, Stevens ME, Mohandas N, et al Transgenic knockout mice with exclusively human sickle hemoglobin and sickle cell disease. Science. 1997;278: 876–878. 9346488 10.1126/science.278.5339.876

[18] Hsu LL, Champion HC, Campbell-Lee SA, Bivalacqua TJ, Manci EA, Diwan BA, et al Hemolysis in sickle cell mice causes pulmonary hypertension due to global impairment in nitric oxide bioavailability. Blood. 2007;109: 3088–3098. 17158223 10.1182/blood-2006-08-039438 PMC1852224

[19] Fallest PC, Trader GL, Darrow JM, Shupnik MA. Regulation of rat luteinizing hormone B gene expression in transgenic mice by steroids and a gonadotropin-releasing hormone antagonist. Biol Reprod. 1995;53:103–109. 7669840 10.1095/biolreprod53.1.103

[20] Chung JY, Chen H, Midzak A, Burnett AL, Papadopoulos V, Zirkin BR. Drug ligand-induced activation of translocator protein (TSPO) stimulates steroid production by aged brown Norway rat Leydig cells. Endocrinology. 2013;154: 2156–2165. 10.1210/en.2012-2226 23525219 PMC3740486

[21] Salva A, Klinefelter GR, Hardy MP. Purification of rat leydig cells: increased yields after unit-gravity sedimentation of collagenase-dispersed interstitial cells. J Androl. 2001;22: 665–671. 11451364

[22] Hurt KJ, Musicki B, Palese MA. Crone JC, Becker RE, Moriarty Jl, et al Akt-dependent phosphorylation of endothelial nitric oxide synthase mediated penile erection. Proc Natl Acad Sci. USA 2002;99: 4061–4066. 11904450 10.1073/pnas.052712499 PMC122648

[23] Montezano AC, Burger D, Ceravolo GS, Yusuf H, Montero M, Touyz RM. Novel Nox homologues in the vasculature: Focusing on Nox4 and Nox5. Clin Sci (Lond). 2011; 120:131–141. 10.1042/CS20100384 21039341

[24] Loscalzo J. Antioxidant enzyme deficiencies and vascular disease. Expert Rev Endocrinol Metab. 2010;5: 15–18. 20190873 10.1586/eem.09.41 PMC2827847

[25] Catala A. Lipid peroxidation of membrane phospholipids generates hydroxy-alkenals and oxidized phospholipids active in physiological and/or pathological conditions. Chem Phys Lipids. 2009;157: 1–11. 10.1016/j.chemphyslip.2008.09.004 18977338

[26] Musicki B, Bivalacqua TJ, Champion HC, Burnett AL. Sildenafil promotes eNOS activation and inhibits NADPH oxidase in the transgenic sickle cell mouse penis. J Sex Med. 2014;11: 424–430. 10.1111/jsm.12391 24251665 PMC4011711

[27] Williams R, Lemaire P, Lewis P, McDonald FB, Lucking E, Hogan S, et al Chronic intermittent hypoxia increases rat sternohyoid muscle NADPH oxidase expression with attendant modest oxidative stress. Front Physiol. 2015 30; 6:15 10.3389/fphys.2015.00015 25688214 PMC4311627

[28] Le B, Chen H, Zirkin B, Burnett A. New targets for increasing endogenous testosterone production: clinical implications and review of the literature. Andrology. 2014;2: 484–490. 10.1111/j.2047-2927.2014.00225.x 24817562

[29] Papadopoulos V, Liu J, Culty M. Is there a mitochondrial signaling complex facilitating cholesterol import? Mol Cell Endocrinol. 2007;265–266: 59–64. 10.1016/j.mce.2006.12.004 17280776

[30] Rone MB, Fan J, Papadopoulos V. Cholesterol transport in steroid biosynthesis: role of protein-protein interactions and implications in disease states. Biochim Biophys Acta. 2009;1791: 646–658. 10.1016/j.bbalip.2009.03.001 19286473 PMC2757135

[31] Jaremko L, Jaremko M, Giller K, Becker S, Zweckstetter M. Structure of the mitochondrial translocator protein in complex with a diagnostic ligand. Science. 2014;343: 1363–1366. 10.1126/science.1248725 24653034 PMC5650047

[32] Midzak A, Papadopoulos V. Binding domain-driven intracellular trafficking of sterols for synthesis of steroid hormone, bile acid and oxysterols. Traffic. 2014;15: 895–914. 10.1111/tra.12177 24890942

[33] Bomhard EM, Gelbke HP. Hypoxaemia affects male reproduction: a case study of how to differentiate between primary and secondary hypoxic testicular toxicity due to chemical exposure. Arch Toxicol. 2013;87: 1201–1218. 10.1007/s00204-013-1024-6 23430139

[34] Odie`vre MH, Verger E, Silva-Pinto AC, Elion J. Pathophysiological insights in sickle cell disease. Indian J Med Res. 2011;134: 532–537. 22089617 PMC3237253

[35] Zhang GL, Dai DZ, Zhang C, Dai Y. Apocynin and raisanberine alleviate intermittent hypoxia induced abnormal StAR and 3β-HSD and low testosterone by suppressing endoplasmic reticulum stress and activated p66Shc in rat testes. Reprod Toxicol. 2013;36: 60–70. 10.1016/j.reprotox.2012.12.002 23270704

[36] Cohen G, Riahi Y, Sunda V, Deplano S, Chatgilialoglu C, Ferreri C, et al Signaling properties of 4-hydroxyalkenals formed by lipid peroxidation in diabetes. Free Radic Biol Med. 2013;65: 978–987. 10.1016/j.freeradbiomed.2013.08.163 23973638

[37] Luo L, Chen H, Trush MA, Show MD, Anway MD, Zirkin BR. Aging and the brown Norway rat leydig cell antioxidant defense system. J Androl. 2006;27: 240–247. 16304208 10.2164/jandrol.05075

[38] Reyes JG, Farias JG, Henríquez-Olavarrieta S, Madrid E, Parraga M, Zepeda AB, et al The hypoxic testicle: physiology and pathophysiology. Oxid Med Cell Longev. 2012;2012: 929285 10.1155/2012/929285 23056665 PMC3465913

[39] Hebbel RP, Vercellotti G, Nath KA. A systems biology consideration of the vasculopathy of sickle cell anemia: the need for multi-modality chemo-prophylaxsis. Cardiovasc Hematol Disord Drug Targets. 2009; 9:271–292. 19751187 10.2174/1871529x10909040271 PMC2914570

[40] Collin O, Bergh A, Damber JE, Widmark A. Control of testicular vasomotion by testosterone and tubular factors in rats. J Reprod Fertil. 1993;97: 115–121. 8464001 10.1530/jrf.0.0970115

[41] Holmes NM, Kane CJ. Testicular infarction associated with sickle cell disease. J Urol. 1998;160: 130 9628625

[42] Li M, Fogarty J, Whitney KD, Stone P. Repeated testicular infarction in a patient with sickle cell disease: a possible mechanism for testicular failure. Urology. 2003;62: 551xi–551xiv. 10.1016/s0090-4295(03)00482-5 12946770

[43] Gofrit ON, Rund D, Shapiro A, Pappo O, Landau EH, Pode D. Segmental testicular infarction due to sickle cell disease. J Urol. 1998;160: 835–836. 9720564 10.1016/S0022-5347(01)62803-9

[44] Tajar A, Forti G, O'Neill TW, Lee DM, Silman AJ, Finn JD, et al; EMAS Group. Characteristics of secondary, primary, and compensated hypogonadism in aging men: evidence from the European Male Ageing Study. J Clin Endocrinol Metab. 2010;95: 1810–1818. 10.1210/jc.2009-1796 20173018

[45] Wu FC, Tajar A, Pye SR, Silman AJ, Finn JD, O'Neill TW, et al Hypothalamic-pituitary-testicular axis disruptions in older men are differentially linked to age and modifiable risk factors: the European Male Aging Study. J Clin Endocrinol Metab. 2008;93: 2737–2745. 10.1210/jc.2007-1972 18270261

[46] Huber G, Staub JJ, Meier C, Mitrache C, Guglielmetti M, Huber P, et al Prospective study of the spontaneous course of subclinical hypothyroidism: prognostic value of thyrotropin, thyroid reserve, and thyroid antibodies. J Clin Endocrinol Metab. 2002;87: 3221–3226. 12107228 10.1210/jcem.87.7.8678

[47] Manci EA, Hillery CA, Bodian CA, Zhang ZG, Lutty GA, Coller BS. Pathology of Berkeley sickle cell mice: similarities and differences with human sickle cell disease. Blood. 2006;107: 1651–1658. 16166585 10.1182/blood-2005-07-2839 PMC1895417

[48] Kaul DK, Zhang X, Dasgupta T, Fabry ME. Arginine therapy of transgenic-knockout sickle mice improves microvascular function by reducing non-nitric oxide vasodilators, hemolysis, and oxidative stress. Am J Physiol Heart Circ Physiol. 2008;295: H39–47. 10.1152/ajpheart.00162.2008 18456737 PMC2494769

[49] Ridha A, Khan A, Al-Abayechi S, Puthenveetil V. Acute compartment syndrome secondary to rhabdomyolysis in a sickle cell trait patient. Lancet. 2014;384: 2172 10.1016/S0140-6736(14)61944-9 25497201

[50] Naik RP, Derebail VK, Grams ME, Franceschini N, Auer PL, Peloso GM, et al Association of sickle cell trait with chronic kidney disease and albuminuria in African Americans. JAMA. 2014;312:2115–2125. 10.1001/jama.2014.15063 25393378 PMC4356116

